# Laparoscopic adrenalectomy performed by a general surgeon on functioning adrenal tumors: Treatment outcomes and risk prediction of persistent hypertension

**DOI:** 10.5339/qmj.2024.30

**Published:** 2024-08-08

**Authors:** Thawatchai Tullavardhana

**Affiliations:** 1 Department of Surgery, Faculty of Medicine, Srinakharinwirot University, Ongkharak, Nakhon Nayok, Thailand *Email: thawatchait@g.swu.ac.th

**Keywords:** Adrenal cortical adenoma, adrenal gland diseases, adrenalectomy, laparoscopic surgery, hypertension

## Abstract

**Background:**

Functional adrenal tumors may contribute to poor hypertension control and electrolyte abnormalities, thus increasing the risk of cardiovascular mortality. Currently, laparoscopic adrenalectomy is an effective surgical option that contributes to improved treatment outcomes as compared to open surgery. The purpose of this study was to evaluate the outcomes of laparoscopic adrenalectomy performed by a general surgeon at a low-volume center and to identify clinicopathological risk factors for postoperative persistent hypertension.

**Methods:**

A retrospective study of patients with functional adrenal tumors who underwent laparoscopic adrenalectomy at Srinakharinwirot University, Thailand, between 2014 and 2022. Clinicopathologic and postoperative data were examined.

**Results:**

This study included twenty-five patients; the indications for laparoscopic adrenalectomy included primary aldosteronism in 19 (76%), pheochromocytoma in 4 (16%), and Cushing’s syndrome in 2 (8%). The average time of surgery was 103.5 ± 19.7 min, and intraoperative complications occurred in three patients (12%), with one patient requiring conversion to open surgery (4%). The postoperative systolic (125 ± 15 vs. 158 ± 18 mmHg; *p* < 0.001) and diastolic (78.5 ± 6.7 vs. 95.3 ± 10 mmHg; *p* = 0.013) blood pressure significantly decreased compared to prior surgery, but only 19 patients (76%) achieved a cure for hypertension. Multivariate analysis revealed that the patient’s physical status, as classified by the American Society of Anesthesiologists (odds ratio (OR) = 0.66, 95% confidence interval (CI) 0.43–1.32, *p* = 0.001), and the need for at least three antihypertensive medicines (OR = 0.7, 95% CI 0.36–1.2, *p* = 0.002), were independent predictive factors of persistent hypertension after surgery.

**Conclusion:**

Laparoscopic adrenalectomy is a safe and effective surgical treatment for functional adrenal tumors, even when performed in a low-volume center. According to the American Society of Anesthesiologists’ physical categorization, the patient’s physical condition and the necessity for at least three antihypertensive medications are predictors of postoperative hypertension.

**Trial registration:**

The study was registered with the Thai Clinical Registry Trials: TCTR20230707007.

## Background

The incidence of adrenal incidentaloma was observed in 0.4% of patients who had abdominal computed tomography, with the majority of the tumors being non-functional.^1,2^ Nevertheless, 10%–15% of the cases exhibited functioning adrenal tumors. Excessive production of hormones, including catecholamines and aldosterone, can result in inadequate control of hypertension. Similarly, imbalances in electrolytes, such as low levels of potassium caused by aldosterone and cortisol, can also contribute to poor hypertension management. These variables may elevate the risk of developing coronary artery disease, cardiac arrhythmia, muscular weakness, and stroke.^3,4^

Most functioning adrenal gland tumors are caused by: 1) Cushing’s syndrome, with a 6.4% incidence, which is suspected if the cortisol level is greater than 5 mg/dL and validated by an overnight 1 mg dexamethasone suppression test; 2) Pheochromocytoma, a catecholamine-producing tumor with a reported incidence of 3.1%, for which a rise in plasma metanephrines and normetanephrine supports the diagnosis; and 3) Primary hyperaldosteronism, a disease with an incidence of 0.6% that causes excessive aldosterone production. Plasma aldosterone levels over 15 ng/dL support the diagnosis.^5-7^

Currently, laparoscopic adrenalectomy is the most prevalent therapeutic approach for individuals affected by adrenal tumors, encompassing both functional and non-functional variants. Previous studies showed that postoperative complications were less than 10%,^8^ while the open adrenalectomy technique showed a significantly higher incidence of overall postoperative complications, reaching 27%.^9^ The use of laparoscopic adrenalectomy may not be recommended for tumors over 10 cm in size, as well as in situations where adrenal malignancy is suspected, leading to a higher potential for postoperative hemorrhage.^10^

Given that the author institute serves as a referral center for a rural hospital in eastern Thailand, it specializes in the treatment of functional adrenal tumors. Furthermore, there has been a lack of previous research on predicting the risk of persistent hypertension after surgery. Thus, the primary objective of this study was to examine the results of a laparoscopic adrenalectomy conducted by a general surgeon in a medium-sized university hospital in rural Thailand, focusing on the management of hypertension and hypokalemia. The second objective was to identify clinicopathological risk factors for predicting postoperative persistent hypertension.

## Material and Methods

A retrospective single-center, single surgeon, cross-sectional study of patients with functioning adrenal tumors who underwent laparoscopic adrenalectomy procedures at the Department of Surgery, Faculty of Medicine, Srinakharinwirot University, Thailand, between January 1, 2014 and December 31, 2022, was conducted. The method of research was approved by the institutional ethics committee of Srinakharinwirot University (Ethics code: SWU/EC/M-043/2565). Patient information was gathered in accordance with the Standards for the Protection of Personally Identifiable Health Information.

For potential recruitment, the patients’ information was extracted from the institution’s computerized database between January 1 and May 31, 2023, using the search terms “primary hyperaldosteronism,” “pheochromocytoma,” “Cushing’s syndrome,” “adrenal gland tumor,” and “adrenalectomy.” In accordance with the Standards for the Protection of Personally Identifiable Health Information, patient information was obtained.

### Preoperative evaluation

Plasma aldosterone levels and plasma renin activity were used to screen patients suspected of primary hyperaldosteronism, and a saline loading test was used to confirm the diagnosis. Plasma and urine metanephrine were used to confirm a pheochromocytoma diagnosis, whereas serum cortisol, adrenocorticotropic hormone (ACTH), and dexamethasone suppression tests were applied to verify a Cushing’s disease diagnosis.

The size, location, and characteristics of the adrenal adenoma were routinely assessed using a computed tomography scan of the abdomen that was conducted in all patients.

Nonetheless, since the institute’s adrenal venous sample test became available in 2016, only ten patients (52.6% of the primary hyperaldosteronism patients) had undergone the test to distinguish between unilateral and bilateral disease. The preoperative assessment revealed the presence of unilateral disease in all patients.

Prior to the surgical procedure, all patients were evaluated for physical wellness and underwent consultation with an endocrinologist. The laparoscopic adrenalectomy procedures were conducted utilizing a transperitoneal route, accessing through the flank, while the patient was positioned in the lateral decubitus posture, as previously described in published literature.^11^

The following data were collected and prepared for analysis in the Microsoft Excel spreadsheet program (Microsoft Corporation, USA): patient characteristics, comorbidities, blood pressure, blood chemistry, hormonal status, radiologic findings, surgical intervention, surgical findings, postoperative outcomes (including postoperative complications, hypertension controls, correction of hypokalemia, and length of stay), and pathologic results (including adrenal gland size and weight, tumor size, and the pathologic diagnosis).

The study’s inclusion criteria were: 1) patients diagnosed with functioning adrenal tumors. 2) underwent laparoscopic adrenalectomy; and 3) aged between 18 and 80 years.

Furthermore, this study included patients who were categorized according to the American Society of Anesthesia (ASA) physical status classification. Class I consisted of patients in a normal, healthy state; Class II included patients with mild systemic disease; and Class III comprised patients with severe systemic disease. This study, however, excludes participants with nonfunctional adrenal tumors, metastasized adrenal tumors, or suspected adrenal malignancies.

The patients were followed up 14–30 days after surgery to assess postoperative complications and then every 3 months to monitor their blood pressure and significant cardiovascular-related complications (including myocardial infarction and stroke) and mortality.

Persistent hypertension was defined as systolic and diastolic blood pressures greater than 140 and 90 mmHg, respectively, after 1 month following surgery and requiring anti-hypertensive drugs for blood pressure control.

### Statistical analysis

The statistical analysis was conducted using version 27.0 of the Statistics Package for Social Sciences (SPSS), developed by SPSS Inc. in Chicago, IL, USA. A descriptive analysis was used to assess the continually gathered data, encompassing clinical and demographic characteristics. The chi-square, Student’s t-test, and one-way ANOVA were used to examine data that varied in terms of categories and continuous variables.

To investigate the crucial role of clinicopathologic parameters in predicting postoperative persistent hypertension, a two-way univariate analysis and multiple logistic regression analysis were performed. A p-value of less than 0.05 was considered statistically significant.

## Results

A total of 33 patients underwent laparoscopic adrenalectomy, but 8 patients were ruled out since there were 5 non-functional tumors, 2 malignant adrenal tumors, and 1 metastatic tumor. As a result, 25 patients were included in this study, with a mean age of 45.36 ± 5.6 years (range: 24–73 years). The vast majority of patients were female (76%). Indications for laparoscopic adrenalectomy included primary aldosteronism in 19 patients (76%), pheochromocytoma in 4 patients (16%), and Cushing’s syndrome in 2 patients (8%).

All of the patients exhibited hypertension, with 88% experiencing poorly controlled hypertension and 22% developing hypertension at a young age. The other clinical manifestations were hypokalemia (84%) and proximal muscular weakness (28%). ASA physical status classifications II and III were found in 64% and 36% of patients, respectively, and previous abdominal surgery was found in 4 patients (2 gynecologic surgeries, 1 appendectomy, and 1 laparoscopic cholecystectomy). The patients’ demographic data are illustrated in Table 1.

The author’s institution usually uses doxazocin (an alpha blocker), amlodipine, verapamil (a calcium channel blocker), atenolol (a beta blocker), enalapril (angiotensin-converting enzyme inhibitors), and hydralazine (a direct vasodilator) as antihypertensive drugs to effectively manage blood pressure in patients diagnosed with functioning adrenal tumors.

Nineteen patients (76%) underwent left adrenalectomy, while the remaining patients underwent right adrenalectomy. The mean operative duration was 103.5 ± 19.7 min (range: 55–185 min), and the average intraoperative blood loss was 80.6 ± 49.17 mL (range: 5–1200 mL). Injury to the inferior vena cava (IVC) was found in one patient, causing massive bleeding and requiring a conversion to open surgery, as illustrated in Table 2. The patient required two units of blood transfusions, and the postoperative period was uneventful. One patient had a urinary tract infection after surgery, which can be managed with oral medications. The average length of stay was 2.2 ± 0.2 days (ranging from 1 to 5 days), as illustrated in Table 3.

Other intraoperative complications included injuries to the left renal artery (1 case) and the right diaphragm (1 case), both of which can be successfully repaired by laparoscopic surgery without postoperative sequelae. There was no mortality in this study. The average adrenal gland size was 5.35 ± 0.3-mm^3^ (range: 3–9-mm^3^), with adrenocortical adenomas accounting for 92% of pathologic diagnoses.

Laparoscopic adrenalectomy cured hypertension in 19 patients (76%) by significantly lowering both mean postoperative systolic (125 ± 15 vs. 158 ± 18 mmHg; *p* < 0.001) and mean diastolic blood pressure (78.5 ± 6.7 vs. 95.3 ± 10 mmHg; *p* = 0.013) compared to prior to surgery. The postoperative plasma aldosterone level was decreased significantly in hyperaldosteronism patients (113.1 ± 38 vs. 1769.3 ± 70 ng/dL; *p* = 0.014), and all the patients were cured of hypokalemia, as evidenced by a significant increase in serum potassium levels after surgery (3.7 ± 0.3 vs. 2.9 ± 0.2; *p* < 0.001), as illustrated in Table 4.

Persistent hypertension was discovered in six patients (24%), necessitating the use of anti-hypertensive drugs to control blood pressure. In a univariate analysis, age (*p* = 0.049), body mass index (*p* = 0.002), ASA classification (*p* = 0.004), diabetes mellitus (*p* = 0.004), preoperative systolic blood pressure (*p* = 0.007), requirement for at least three anti-hypertensive drugs for blood pressure control (*p* < 0.001), and gland weight (*p* = 0.046) appeared to be risk variables for persistent hypertension.

Nevertheless, there was no significant association between the occurrence of postoperative persistent hypertension and patient sex (*p* = 0.094), pre-operative diastolic blood pressure (*p* = 0.987), gland size (*p* = 0.952), tumor size (*p* = 0.918), or the diagnosis of a functional adrenal tumor. Regarding the primary hyperaldosteronism group, all patients experienced a significant decrease in postoperative blood aldosterone levels. However, 10.5% (2 out of 19 patients) remained experiencing hypertension after the surgery. This clarified the finding that serum aldosterone levels did not provide a risk for persistent hypertension (*p* = 0.776).

A multiple logistic regression analysis, however, revealed that ASA classification (odds ratio (OR) = 0.66, 95% confidence interval (CI) 0.43–1.32, *p* = 0.001) and requirement for at least three anti-hypertensive drugs for blood pressure control (OR = 0.7, 95% CI 0.36–1.2, *p* = 0.002) were independent risk variables for predicting persistent hypertension, as shown in Table 5.

The average length of follow-up was 18.9 ± 3.1 months (range: 3–53 months), and it was observed that two patients with hyperaldosteronism had recurrent hypertension and only needed one medication to control their blood pressure. During the follow-up period, there were no serious cardiovascular problems such as myocardial infarction, stroke, or cardiovascular-related mortality.

## Discussion

Currently, minimally invasive adrenal surgery includes laparoscopic adrenalectomy via transperitoneal or posterior retroperitoneal approaches, and robotic adrenalectomy has become the standard treatment for functional adrenal tumors due to superior postoperative outcomes compared to open surgery.^12,13^ Since the author’s institution is a medium-sized referral hospital in rural Thailand, general surgeons must play an important role in the surgical management of patients with functional adrenal tumors; thus, all adrenalectomies are performed utilizing a laparoscopic transperitoneal method.

The laparoscopic transperitoneal approach is appropriate for general surgeons with limited laparoscopic adrenalectomy experience for the following reasons: 1) a shorter learning curve; 2) familiarity with laparoscopy views; 3) comfort with intraperitoneal anatomical landmarks; and 4) a large working space to resect large adrenal tumors.^14,15^

The study’s findings reveal that three patients (12%) had an intraoperative complication, two of whom successfully managed it with laparoscopic repairs and had no postoperative sequelae; the other patient (4%) needed to convert to open surgery to repair the IVC and required postoperative blood transfusions. There was no mortality in this study. These findings were consistent with Pang et al.’s study, which reported a 3.2% conversion rate to open surgery and an 8.0% postoperative morbidity rate for laparoscopic adrenalectomy in functional adrenal tumors.^16^

The fact that a single surgeon, who performs over 50 laparoscopic procedures annually, performed the laparoscopic adrenalectomy may have lowered the procedure’s learning curve. Thus, these findings confirmed the safety of laparoscopic adrenalectomy procedures, even when performed in a low-volume center.

The study demonstrated that the postoperative systolic and diastolic blood pressures were significantly lower than preoperatively, and the hypertension cure rate was 76%. This rate is comparable to previous studies that revealed hypertension cure rates in functional adrenal tumors of 43.7–61.3%, 61.5%, and 69%, respectively, in primary hyperaldosteronism, Cushing’s syndrome, and pheochromocytoma.^17-19^

Despite the study’s provision of an adequate follow-up period for postoperative blood pressure monitoring, the small sample size may have limitations on the interpretation of the findings. Furthermore, all patients with primary hyperaldosteronism were free of hypokalemia, which is the result of a significantly decreased serum aldosterone level after surgery.

In the management of poor hypertension, the combination of two antihypertensive drugs (such as angiotensin-converting enzyme inhibitors or angiotensin receptor blockers with calcium channel blockers or beta-blockers) is 2–5 times more effective in lowering blood pressure and may reduce cardiovascular complications compared to increasing the dose of a single drug, such as acute coronary syndrome (29% vs. 40%) and stroke (40% vs. 54%).^20,21^

However, the patients exhibiting resistant hypertension, which is characterized by inadequate regulation of blood pressure despite the use of three anti-hypertensive medications at doses that are well-tolerated, are commonly associated with secondary factors such as obstructive sleep apnea, renovascular disease, and functional adrenal tumors. This condition serves as a prognostic indicator for unfavorable outcomes, including an increase in mortality rates associated with cardiovascular events.^22,23^

Postoperative persistent hypertension was found in 24% of the patients. The cause of persistent hypertension is still unknown. A univariate analysis demonstrated that age, BMI, ASA classification, diabetes mellitus, preoperative systolic pressure, the requirement for at least three anti-hypertensive drugs, and gland weight were associated with risk variables for persistent hypertension.

However, a multivariate analysis demonstrates that the ASA classification and the requirement for at least three anti-hypertensive drugs are independent predictors of postoperative persistent hypertension. This contrasts with previous studies that demonstrated that age and gland weight were independent factors predicting hypertension after surgery.^16,24^

The differences between the risk prediction of persistent hypertension and previous research could be explained as follows: 1) The previous study operated on a larger tumor (mean gland size of 50–60 mm), which may have had a high degree of cortical hyperplasia; 2) The patient’s age (> 60 years) may have had several risk factors for hypertension, including smoking, alcohol abuse, and diabetes mellitus, increasing the risk of cardiovascular death.^25,26^ However, the average age in this study was 45.4 years. 3) This study found that 20% of patients required at least three antihypertensive drugs, which had not been reported in previous studies.

Furthermore, chronic essential hypertension may cause chronic vasoconstrictive and vascular remodeling, which can contribute to increased vascular resistance and persistent hypertension following surgery. Nonetheless, the duration of hypertension has been excluded from the study analysis due to a lack of data.

The current study has limitations as follows: 1) it was a retrospective analysis, and 2) because the incidence of functional adrenal tumors is uncommon in rural Thailand, only a limited number of cases could be examined. Due to the small sample size, this may have a positive effect on the analysis. Additionally, the majority of cases were diagnosed with primary hyperaldosteronism, whereas in Western countries, the majority of cases are cortisol-producing adenomas, which may introduce the potential for a risk of bias. However, a suitable period for postoperative monitoring (average 18.9 months) and biochemical tests comparing preoperative and postoperative results may improve the reliability of the outcomes.

The study’s findings supported the concept that laparoscopic adrenalectomy through the transperitoneal approach performed by a general surgeon is a safe and effective technique in the management of functional adrenal tumors. Additionally, these findings are useful in predicting outcomes in patients who have clinicopathologic parameters that are risk factors for persistent hypertension and should have their blood pressure closely monitored after surgery.

## Conclusion

Laparoscopic adrenalectomy is a safe and effective surgical treatment for functional adrenal tumors in terms of hypertension control, even when performed by a general surgeon in a low-volume center. Independent predictors of postoperative persistent hypertension are the ASA classification and the requirement for at least three anti-hypertensive drugs.

## Conflict of Interest Statement

The authors have no conflicts of interest to declare.

## Authors’ Contributions

TT is responsible for the study design, data acquisition, data analysis and interpretation, and manuscript writing.

## Acknowledgments

The author expresses gratitude to Associate Professor Prinya Akranurakkul from the Department of Surgery, Faculty of Medicine, Srinakharinwirot University, for his guidance and support throughout the research work.

## Ethical Statements

This research was approved by the institutional ethics committee of Srinakharinwirot University (Ethics code: SWU/EC/M-043/2565).

## Figures and Tables

**Table 1.** Illustrates the demographic data of patients.

**Number of patients**	25
**Mean age (years)**	45.36 ± 5.6 (24–73)
**Sex (male:female)**	6:19
**Body mass index** (BMI: kg/m^2^)	25.8 ± 0.96 (16.65–32.82)
**Diagnosis of functional adrenal tumors**
Primary hyperaldosteronism	19 (76%)
Pheochromocytoma	4 (16%)
Cushing’s syndrome	2 (8%)
**Clinical presentation**	
Hypertension	25 (100%)
In the young	12 (48%)
Uncontrol	22 (88%)
Hypokalemia	21 (84%)
Proximal muscle weakness	7 (28%)
**ASA classification**
1	-
2	16 (64%)
3	9 (36%)
**Co-morbidities**	
Diabetes mellitus	6 (24%)
Dyslipidemia	8 (32%)
Cardiovascular disease	2 (8%)
Previous abdominal surgery	4 (16%)
**Anti-hypertensive drugs**	
Required < 3 medications	20 (80%)
Require at least 3 medications	5 (20%)
Follow-up periods (months)	18.9 ± 3.1 (3–53)

ASA classification, American Society of Anesthesia physical status classification.

**Table 2.** Details of the surgical procedure, intraoperative complications, and pathological findings.

**Laparoscopic adrenalectomy**	
Right	6 (24%)
Left	19 (76%)
Conversion to open surgery	1 (4%)
**Operative time (min)**	103.5 ± 19.7 (55–185)
**Estimated blood loss (milliliters: mL)**	80.64 ± 9.17 (5–1200)
**Intraoperative complication**	
Injury to great vessel	2 (8%)
Diaphragmatic injury	1 (4%)
**Pathologic diagnosis**	
Gland size (millimeter: mm)	5.35 ± 0.3 (3–9)
Tumor size (millimeter: mm)	2.05 ± 0.2 (1.1–5)
Gland weight (g)	13 ± 1.2 (5.06–33.5)
Adrenocortical adenoma	23 (92%)
Cortical nodular hyperplasia	2 (8%)

**Table 3.** Postoperative complications and outcomes.

**Postoperative complication**	
Blood transfusion	1 (4%)
Urinary tract infection	1 (4%)
**Postoperative control of hypertension**	
Cure of hypertension	19 (76%)
Recurrent of hypertension (within 2 years)	2 (10.5%)
Persistent hypertension	6 (24%)
**Hypokalemia**	
Cure of hypokalemia	21 (100%)
**Length of stay (days)**	2.2 ± 0.2 (1–5)

**Table 4.** Comparison of pre- and postoperative blood pressure and blood chemistry.

**Outcomes**	**Preoperative**	**Postoperative**	***p* value**
Mean Systolic pressure (mmHg)	158 ± 18	125 ± 15	< 0.001*
Mean Diastolic pressure (mmHg)	95.3 ± 10	78.5 ± 6.7	0.013*
Primary hyperaldosteronism group	1769.3 ± 70	113.1 ± 38	0.014*
• Serum aldosterone level (ng/dL)			
• Serum potassium level (mmol/L)	2.9 ± 0.2	3.7 ± 0.3	< 0.001*

* Statistically significant.

**Table 5.** Predictive risk factors for postoperative persistent hypertension.

	**Univariate analysis**	**Multivariate analysis**	
**Variables**	**(*p*-value)**	**Odds ratio (95% CI)**	***p*-value**
Age	0.049*	−0.24 (−0.0–0.1)	0.210
Sex	0.094		
Primary hyperaldosteronism	0.599		
Phreochromocytoma	0.199		
Cushing’s syndrome	0.429		
Body mass index	0.002*	0.18 (−0.02–0.05)	0.324
ASA classification	0.004*	0.66 (0.43–1.32)	0.001*
Diabetes mellitus	0.004*	−0.1 (−0.71–0.69)	0.977
Preoperative systolic pressure	0.007*	−0.12 (−0.02–0.01)	0.479
Preoperative diastolic pressure	0.987		
Require at least three medicines	< 0.001*	0.7 (0.36–1.2)	0.002*
Gland weight	0.046*	0.15 (−0.03–0.06)	0.453
Gland size	0.952		
Tumor size	0.918		
Preoperative aldosterone level	0.776		

*Statistically significant.

95% CI, 95% confidence interval; ASA classification, American Society of Anesthesia physical status classification.
